# Fuel for Thought? A Systematic Review of Neuroimaging Studies into Glucose Enhancement of Cognitive Performance

**DOI:** 10.1007/s11065-020-09431-x

**Published:** 2020-03-11

**Authors:** Riccarda Peters, David White, Carlee Cleeland, Andrew Scholey

**Affiliations:** Centre for Human Psychopharmacology, Swinburne University of Technology, Melbourne, VIC 3122 Australia

**Keywords:** Neuroimaging, Neurophysiology, Memory, Cognition, Attention, fMRI, fNIRS, EEG, ERP

## Abstract

A transient improvement in cognitive performance can be observed following the ingestion of a glucose drink, a phenomenon known as the ‘glucose facilitation effect’. The effect has been studied thoroughly in the last three decades, but its neural underpinnings remain a matter of speculation. A systematic review was conducted to evaluate the current evidence from studies applying neuroimaging or neurophysiological methods to investigate the glucose enhancement effect. Eleven studies met the inclusion criteria of using neuroimaging in conjunction with cognitive outcomes. Six studies employed electroencephalography (EEG), four used functional magnetic resonance imaging (fMRI) and one employed functional near-infrared spectroscopy (fNIRS). All but one study reported modulation of neurophysiology or neuroimaging markers following glucose, while only five studies reported significant changes in cognitive outcomes. The evidence suggests that glucose administration enhances neurocognitive markers of episodic memory and attentional processes underpinned by medial temporal and frontal activation, sometimes in the absence of measurable behavioural effects. Further exploration of glucose facilitation using neuroimaging measures with increased sample sizes is warranted to replicate these findings.

## Introduction

The brain is the most metabolically active organ in the human body, consuming around 25% of available glucose under resting conditions (Magistretti, 1999). Increasing systemic glucose levels has been shown to improve cognitive performance. More than three decades of research in this area has demonstrated that the simple administration of supplemental glucose, can improve cognitive performance during the time that the glucose levels are elevated (for meta-analysis/review see Messier, 2004; Riby, 2004; Smith et al., 2011). This transient improvement in cognitive performance has been termed the ‘glucose facilitation effect’ (Smith et al., 2011).

Typically, studies investigating the glucose facilitation effect involve the administration of a glucose drink (usually 25 g or 50 g pure powdered glucose dissolved in water), as well as a taste and appearance matched placebo drink with an artificial sweetener (e.g. saccharine or aspartame). Since the glucose facilitation effect is transient, it lends itself to repeated-measures designs, where participants attend the laboratory on two occasions, receiving the glucose and the placebo drink in a counterbalanced order. Between-subjects designs, where participants attend the laboratory once and are assigned randomly to either ‘glucose’ or ‘placebo’ condition, are less common. Most studies of glucose facilitation require participants to attend testing sessions in the morning after an overnight fast. In healthy individuals the concentration of blood glucose peaks after approximately 30 min following ingestion and returns back to baseline after 2 h or so (Macpherson et al., 2015). In most studies, cognitive testing takes place between 10 min and 2 h following ingestion. Capillary blood glucose is typically monitored with a finger prick at baseline (pre-dose) and at pre-determined intervals after treatment, to ensure that glucose treatment effectively modulated blood glucose (Smith et al., 2011).

Glucose facilitation of cognitive performance has been demonstrated in a range of populations including children (Benton & Stevens, 2008; Horne, Barr, Valiante, Zelazo, & Young, 2006), adolescents (Smith & Foster, 2008), healthy adults (e.g. Owen, Scholey, Finnegan, and Sünram-Lea (2013) and older adults (Macpherson et al., 2015: for general reviews see Riby, 2004; van der Zwaluw, van de Rest, Kessels, & de Groot, 2015). The effect has also been demonstrated in clinical cohorts such as schizophrenia (Stone, Seidman, Wojcik, & Green, 2003), mild cognitive impairment (Riby et al., 2009) and dementia (Manning, Ragozzino, & Gold, 1993). The glucose facilitation effect has been most often reported for long-term memory measures (see Smith et al., 2011). Although improvements have also been observed across a range of cognitive domains (for review see Hoyland, Lawton, & Dye, 2008; Messier, 2004; Riby, 2004; Riby & Riby, 2006; van der Zwaluw et al., 2015). These include central processing speed and reaction times (Benton, Owens, & Parker, 1994), working memory performance (Kennedy & Scholey, 2000; Scholey, Harper, & Kennedy, 2001), executive function (Brandt, Gibson, & Rackie, 2013), problem solving (Miller, Bourrasseau, & Blampain, 2013) and attention (Messier, Gagnon, & Knott, 1997).

Despite numerous studies into the impact of increased blood glucose on cognitive tasks, the neurocognitive mechanisms as well as the precise cognitive abilities susceptible to the glucose facilitation effect remain unclear. On the one hand, it has been argued that the level of mental demand during cognitive processing is important in determining task sensitivity to glucose facilitation. Tasks that require higher levels of mental effort tend to recruit more neural resources, and therefore require more metabolic substrate (e.g. glucose and oxygen), to meet cognitive goals (Scholey, 2001; Scholey, Benson, Sela-Venter, Mackus, & Moss, 2019; Scholey, Laing, & Kennedy, 2006; Scholey et al., 2001). More demanding tasks are therefore thought to involve greater glucose utilization and are thus more susceptible to systemic glucose manipulations. Converging evidence suggests that glucose may preferentially enhances cognition under conditions of increased task demand or ‘mental effort’. These include tasks with a higher computational load (Kennedy & Scholey, 2000; Scholey et al., 2001) or those which draw heavily on cognitive resources via divided attention or dual task processing (Macpherson et al., 2015; Scholey, Sünram-Lea, Greer, Elliott, & Kennedy, 2009; Sünram-Lea, Foster, Durlach, & Perez, 2002). These findings suggest that the glucose enhancement effect may have little domain specificity. Regarding neuroanatomical substrates, those tasks which require greater attentional resources and cognitive control tend to be dependent on frontal regions. It has been suggested that these may be implicated in the glucose facilitation effect (Gailliot & Baumeister, 2007; Sünram-Lea & Owen, 2017).

Regarding the functional neuroanatomy of glucose facilitation, the hippocampus has been a major research focus. This is partially based on the association between the apparent differential effects of glucose on tasks thought to be reliant on hippocampal function (Smith et al., 2011; Sünram-Lea, Dewhurst, & Foster, 2008), including verbal episodic memory (Riby, 2004; Smith et al., 2011). The notion that the hippocampus is especially targeted by glucose in mediating memory has gained some support from animal studies investigating the mechanisms of the effect. The hippocampus is densely populated with insulin receptors known to promote cellular glucose uptake (Craft & Watson, 2004; Dore, Kar, Rowe, & Quirion, 1997) and is further enriched with insulin-sensitive glucose transporters (GLUT isoforms) such as GLUT4 (McEwen & Reagan, 2004). Glucose may increase the synthesis of certain neurotransmitters, including hippocampal synthesis of acetylcholine (Messier, 2004). On the other hand studies aimed specifically at differentiating hippocampal and non-hippocampal effects of glucose have shown mixed results (Scholey et al., 2013; Sünram-Lea et al., 2008).

Currently, then, there is some evidence to support a preferential hippocampus-mediated mechanism underlying the glucose facilitation effect, but also a demand-based neurocognitive mechanism. It is worth noting that the two models are not mutually exclusive and it may be that both hippocampal tasks and those which engender higher levels of cognitive demand are both susceptible to glucose facilitation. Studies using neuroimaging and neurophysiology methods have the potential to help elucidate the effect of glucose on the brain and cognition. A growing body of research is using neuroimaging techniques in order to establish the neural substrates of the glucose facilitation effect. To date, however, there has been no systematic evaluation of these studies investigating brain activation patterns related to the effect of glucose on cognition. Therefore, the aim of the present review is to apply systematic review methodology to evaluate the evidence of the acute effects of glucose on neuroimaging measures of brain function and cognitive performance. Studies from all neuroimaging methods were considered and reviewed if appropriate. Methodological considerations are also discussed.

## Methods

The systematic review was conducted according to the PRISMA guidelines for systematic review and meta-analysis protocols (Moher et al., 2009).

### Literature Search

The article search was conducted in PubMed and Web of Science between earliest record and December 1st 2018.

### Search Strategy and Terms

The following search terms were combined using the Boolean operators “AND” and “OR.” Terms were nested by being enclosed in parentheses. Words beginning with “cognit” and “electroence” were truncated by the asterisk (*). The search terms in both databases were as follows: “glucose” AND (“administration” OR “ingestion” OR “intake” OR “facilitation” OR “acute”) AND (“cognit*” OR “performance” OR “memory” OR “executive function” OR “attention” OR “brain” OR “psychological test”) AND (“neuroimaging” OR “brain activity” OR “fMRI” OR “functional magnetic resonance imaging” OR “BOLD” OR “blood oxygen level dependent” OR “EEG” OR “electroence*” OR “event-related potential” OR “ERP” OR “Magnetoencephalography” OR “MEG” OR “near-infrared” OR “NIRS”). The search was limited to human studies and studies published in English.

In Web of Science, search terms were refined by categories (neurosciences OR clinical neurology OR psychiatry OR pharmacology pharmacy OR neuroimaging OR behavioural sciences OR psychology OR nutrition dietetics OR physiology OR psychology biological OR psychology experimental OR medicine research experimental OR food science technology OR psychology clinical OR psychology multidisciplinary OR integrative complementary medicine) AND document type (= article).

**Fig. 1:**
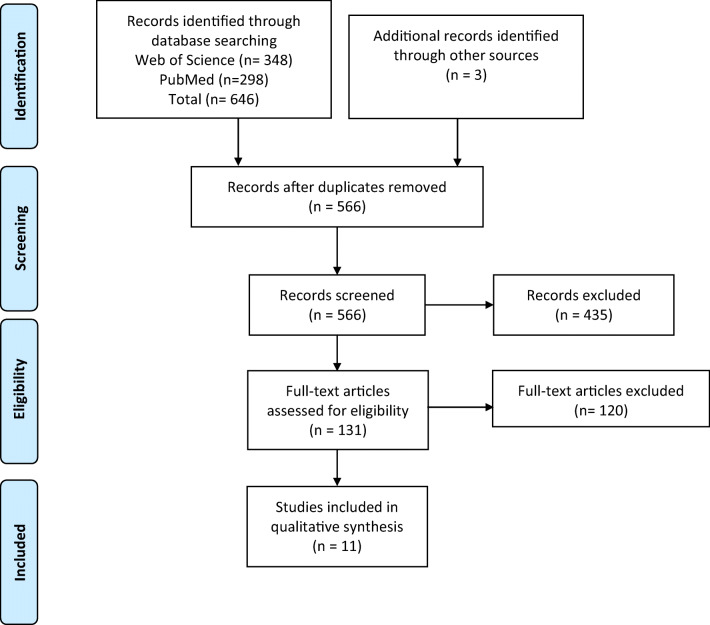
Flowchart of the literature selection procedure

Titles, abstracts and key words were examined first. Full texts were independently reviewed by two authors, with conflicts resolved by a third. Reference lists of identified studies were also searched. Figure 1 summarizes the search process.

### Inclusion Criteria

Human studies of any age group, gender, sample size or sample population were included. Only research into acute oral glucose administration, as an independent variable and as central manipulation were included. Further, studies were only included if they included an appropriate placebo or control condition compared to glucose and if participants had to fast before testing sessions. Studies had to include both a non-invasive neurophysiological or neuroimaging modality as well as measures of cognitive performance.

### Exclusion Criteria

Studies in which glucose was not manipulated were excluded, as well as studies in which there was no pure glucose arm (glucose simultaneously administered with any other study compound, excluding vehicle). Studies were excluded if they only reported cognitive outcomes without neuroimaging measures or vice versa. Studies using ^18^F-fluorodeoxyglucose (FDG) PET imaging were excluded, as FDG PET is a neuroimaging measure which involves administration of the glucose analog as a matter of course.

### Quality Rating

The studies included in the review were analyzed for methodological quality using a modified version of the Jadad Scale (Sarris & Byrne, 2011), which assesses randomization, blinding and reported withdrawals. The modified version used for this review assesses whether 1) the study is described as randomized, 2) the randomization protocol was detailed and appropriate, 3) the study was described as double blind, 4) the blinding process was detailed and appropriate, 5) the study had a control group, 6) the control was detailed and appropriate, 7) there were adequate exclusion criteria, 8) the amount of glucose administered was documented, 9) there was a description of withdrawals and dropouts, and 10) whether the data were reported clearly and adequately. The studies were rated based on these ten items, with a point awarded for each. The total score was obtained by adding the numbers of ratings together, leading to a maximum possible rating of ten points. It follows that a higher score means better quality. Note that criterion 8 has been modified from “Was the intervention at a therapeutic dose?” to “Was the amount administered documented?”, as in Scholey and Owen (2013).

Further, to ensure unambiguous interpretation we specified a number of essential criteria to receive an affirmative rating on item number 6 and 7. The following controls were judged as suitable: taste-matched drink mixed with aspartame or saccharin. In order to study the effect of glucose on cognition and brain function, placebo effects need to be ruled out (item number 6). For item number 7 the following exclusion criteria had to be reported to receive an affirmative answer: diabetes and abnormal fasting blood glucose levels. Authors RP and CC independently rated the studies and cross-compared answers. In case of disagreement conflicts were resolved by DW.

### Synthesis

Extracted information included year of publication, country, participant demographics including number of subjects and number of subjects included in analysis, age, gender, amount of glucose administered, research design, amount of placebo administered, duration of fasting before testing, frequency of blood glucose monitoring, imaging modality and analysis mode, cognitive outcome measure, results of cognitive task and neuroimaging or neurophysiology measures. Due to the relatively small number of studies identified by the search, and the heterogeneity of methods and outcomes assessed, meta-analysis was not possible nor appropriate.

## Results

The initial database search captured a total of 646 studies, 348 studies were found in the Web of Science database and 298 in PubMed. Three studies were identified from reference lists in other articles captured in the search. After the removal of duplicates, 566 studies were examined against the inclusion and exclusion criteria. A flowchart of the selection procedure is shown in Fig. 1.

Eleven studies met the inclusion criteria for this review, their characteristics are summarised in Tables 1 and 2. The studies were conducted in several countries including UK (*n* = 3), Canada (*n* = 2), USA (n = 2), Australia (*n* = 1), Spain (n = 1), Switzerland (n = 1) and Korea (n = 1). Six studies were conducted with healthy adult participants (An, Jung, Kim, Lee, & Kim, 2015; Brown & Riby, 2013; Parent et al., 2011; Riby et al., 2008; Serra-Grabulosa, Adan, Falcon, & Bargallo, 2010; Zanchi et al., 2018). Three studies were conducted with healthy elderly samples (Gagnon et al., 2012; Knott, Messier, Mahoney, & Gagnon, 2001; Scholey et al., 2015). One study was conducted with healthy adolescent participants (Smith et al., 2009) and one study was conducted with adults diagnosed with chronic schizophrenia (Stone, Thermenos, Tarbox, Poldrack, & Seidman, 2005).

**Table 1:** Summary of studies included in the systematic review which used electroencephalography (EEG). Including evoked response potential (ERP) components. WS = Within-subject, repeated measures (crossover) design; BS = Between-subject design; N = Number of participants; ↑ or ↓ indicates glucose-related improvement or decrease, respectively; ACC = Anterior cingulate; DLPFC = Dorsolateral prefrontal cortex; FPN = Frontoparietal Network; SN = Salience Network; QR = Quality rating based on modified Jadad Scale. Studies are listed in the order they appear in the manuscript

Reference	Design	N	Participants	Fasting	Glucose	Placebo	Task	Behavioral Results	Neuroimaging Results	QR (/10)
*EEG/ ERP P3b*										
(Riby et al., 2008)	WS	11	Healthy young adults mean age 28.7 ± 6 (5 m/6f)	2 h	25 g	38 mg saccharin	Visual 3-stimulus oddball task	No treatment effect.	**↓** Reduced P3b amplitude	6
(Knott et al., 2001)	WS	10	Elderly subjects mean age 62.7 ± 5.7 (9 m/1f)	overnight fast	50 g glucose +4 mg saccharin	50.6 mg saccharin	Sternberg memory scanning task (assesses speed and efficiency of short-term memory storage and processing)	No treatment effect.	No treatment related effect.	7
*EEG/ ERP LP/ FN400*										
(Brown & Riby, 2013)	BS	18 glucose; 17 placebo	Young adults mean age 22.17 ± 5.97 (14 m/21f)	2 h	25 g	37.5 mg saccharin	Episodic memory (item recognition task, words and pictures); Attention (Stroop) Task	**↑** Increased accuracy during episodic memory	**↑** Enhanced LP effect	7
(Smith et al., 2009)	WS	17	Healthy adolescents mean age 14.4 ± 1.5 (9 m/9f)	2 h	25 g	5 of aspartame tablets	Recognition memory task	**↑** Faster response times	**↑** Enhanced LP; **↑** Enhanced FN400	5
(Scholey et al., 2015)	WS	12	Healthy elderly mean age 69.33 ± 1.69 (7 m/5f)	overnight fast	25 g	30 mg saccharin	Word recognition: remember-know paradigm both with and without psychomotor tracking during auditory presentation of words)	**↓** Reduced overall accuracy	**↑** Enhanced LP effect	8
*EEG/resting state*										
(Knott et al., 2001)	WS	10	Elderly subjects mean age 62.7 ± 5.7 (9 m/1f)	overnight fast	50 g glucose +4 mg saccharin	50.6 mg saccharin	Eyes closed, 6 min vigilance-controlled EEG acquisition	No treatment effect.	No treatment related effect.	7
(An et al., 2015)	WS	24	Healthy adults mean age 33.1 ± 2.6 (11 m/13f)	≥ 8 h	17 g	none	Digit span: Wechsler Adult Intelligence Scale revised; Spatial span test: Corsi block-tapping test	**↑**Improvements in attentional performance	**↑** Increased power in theta and low alpha bands on resting-state EEG	4

**Table 2:** Summary of studies included in the systematic review which used functional magnetic resonance imaging (fMRI) and functional near infrared spectroscopy (fNIRS). WS = Within-subject, repeated measures (crossover) design; BS = Between-subject design; N = Number of participants; ↑ or ↓ indicates glucose-related improvement or decrease, respectively; ACC = Anterior cingulate; DLPFC = Dorsolateral prefrontal cortex; FPN = Frontoparietal Network; SN = Salience Network; QR = Quality rating based on modified Jadad Scale. Studies are listed in the order they appear in the manuscript

Reference	Design	N	Participants	Fasting	Glucose	Placebo	Task	Behavioral Results	Neuroimaging Results	QR (/10)
*fMRI*										
(Stone et al., 2005)	WS	7	Adults diagnosed with chronic schizophrenia, mean age 38.8 ± 10.7 (4 m/3 f)	≥ 8 h	50 g	Saccharin	Recognition memory task (verbal encoding and recognition)	No treatment effect.	**↑** Greater activation of left parahippocampus during novel sentence encoding	7
(Parent et al., 2011)	WS	12	Healthy male adults mean age 24.1 (14 m)	overnight fast	50 g	23.7 mg saccharin	Picture encoding task, free recall, delayed free recall following day	No treatment effect.	**↑** Greater superior parietal sulcus activation during encoding; **↑** Increased regional activations related to later successful subsequent memory**;** **↑** Increased connectivity of hippocampus and amygdala	7
(Serra-Grabulosa et al., 2010)	BS	10	Healthy young adults mean age 19.6 (5 m/5f)	overnight fast	75 g	150 ml water	CPT- IP task (sustained attention task)	No treatment effect.	No treatment related effect.	7
(Zanchi et al., 2018)	WS	12	Healthy young adults mean age 24.8 (12 m)	overnight fast	75 g	300 ml water	N-back task (0-back, 1-back and 2-back); fMRI paradigm: Go/No-Go Task	No treatment effect.	**↓** N-back: decreased activation in frontal areas (ACC, DLPFC); **↓** Go/No-Go: decreased activation in ACC, DLPFC, insula and visual cortex; **↑** Resting state: increased connectivity in FPN and SN	8
*fNIRS*										
(Gagnon et al., 2012)	WS	15	Elderly adults mean age 69.4 (16w/4 m)	overnight fast	50 g	23.7 mg saccharin	Dual Task (visual discrimination)	**↑** Dual task coordination improvements	**↑** Increase in prefrontal activation	7

Seven of the included studies used Electroencephalography (EEG) as a measure of brain activity, five of these focused on event-related potentials (ERPs: Brown & Riby, 2013; Knott et al., 2001; Riby et al., 2008; Smith et al., 2009; Scholey et al., 2015). Two studies reported spectral analysis of resting EEG (An et al., 2015; Knott et al., 2001). Four studies were conducted using task related functional magnetic resonance imaging (fMRI: Parent et al., 2011; Serra-Grabulosa et al., 2010; Stone et al., 2005; Zanchi et al., 2018), of which two also reported measuring fMRI connectivity (Parent et al., 2011; Zanchi et al., 2018). One study was conducted using functional near infrared spectroscopy (fNIRS: Gagnon et al., 2012).

Sample sizes ranged from seven (Stone et al., 2005) to 35 participants (Brown & Riby, 2013). Ten of the studies employed a within-subject repeated measures design in which participants serve as their own control (crossover design), only one used a between-subject design (Brown & Riby, 2013). In one repeated-measures study, testing pre- and post-ingestion was conducted on the same day (Serra-Grabulosa et al., 2010), in all other studies with repeated-measures, testing of the active and placebo drink occurred on separate days. In seven studies participants were asked to attend the lab after at least eight hours of fasting (usually overnight: An et al., 2015; An et al., 2015; Gagnon et al., 2012; Knott et al., 2001; Parent et al., 2011; Serra-Grabulosa et al., 2010; Scholey et al., 2015; Stone et al., 2005; Zanchi et al., 2018) and in three studies participants were instructed to fast for two hours prior to the testing sessions (Brown & Riby, 2013; Riby et al., 2008; Smith et al., 2009).

In ten studies, glucose was administered orally as a drink, in one study the drink was administered via a nasogastric tube (Zanchi et al., 2018). The amount of glucose in the active drink was 25 g in four studies (Brown & Riby, 2013; Riby et al., 2008; Scholey et al., 2015), 50 g in four studies (Gagnon et al., 2012; Knott et al., 2001; Parent et al., 2011; Stone et al., 2005), 75 g in two studies (Serra-Grabulosa et al., 2010; Zanchi et al., 2018) and 17 g in one study (An et al., 2015). Seven studies used the non-caloric sweetener saccharine in the placebo condition (Brown & Riby, 2013; Gagnon et al., 2012; Knott et al., 2001; Parent et al., 2011; Riby et al., 2008; Scholey et al., 2015; Stone et al., 2005), while aspartame was used in one (Smith et al., 2009). In two studies baseline testing was conducted without the administration of any reported drink (An et al., 2015; Serra-Grabulosa et al., 2010). In one study, the placebo condition was the administration of water without the risk of unblinding participants as a result of delivery via nasogastric tube (Zanchi et al., 2018). All studies included measured baseline capillary blood glucose levels pre-drink administration and at varying intervals, but at least at one point, post-ingestion via a fingerprick with a blood glucose monitor. Only two studies reported exclusion of participants exceeding a certain threshold of blood fasting glucose levels, 7.0 and 6.67 mmol/L (126 or 120 mg/dl) respectively (Gagnon et al., 2012; Stone et al., 2005).

### Quality Rating

The studies included in the review were of varying quality, ten out of eleven studies received a rating equal to or more than five out of ten. One study was rated five (Smith et al., 2009), one was rated six (Riby et al., 2008), six studies were rated seven (Brown & Riby, 2013; Gagnon et al., 2012; Knott et al., 2001; Parent et al., 2011; Stone et al., 2005; Serra-Grabulosa et al., 2010) and two studies were rated eight out of ten (Scholey et al., 2015; Zanchi et al., 2018). Only one study received a score of four (An et al., 2015), due to missing information in the report (see Tables 1 and 2). Derivation of the quality ratings can be found in the supplementary online material. Items relating to the description of the randomization procedure were especially problematic. Only one study detailed the randomization protocol (Scholey et al., 2015) and no study outlined the blinding process, even though eight studies described their study as double-blind (Gagnon et al., 2012; Knott et al., 2001; Parent et al., 2011; Riby et al., 2008; Scholey et al., 2015; Stone et al., 2005; Serra-Grabulosa et al., 2010; Zanchi et al., 2018). The following sections summarize the findings from electrophysiological neuroimaging methods (Table 1) and from fMRI and fNIRS studies (Table 2).

### Electroencephalography (EEG)

Five studies assessed glucose modulation of event-related potentials (ERPs), while two measured changes in spectral activity during resting EEG recordings. The studies using ERPs to measure the glucose facilitation effect focused on different components, elicited by a number of different task paradigms. Studies were grouped and discussed according to studied ERP components to allow meaningful comparisons.

#### Event-Related Potential: P300

Two studies investigated the glucose facilitation effect with ERPs focusing on the P300 component (Knott et al., 2001; Riby et al., 2008). The traditional P300 component, also referred to as P3b, is elicited using an ‘oddball’ paradigm, where a sequence of stimuli is presented and the participant needs to discriminate infrequent target events from frequent standard events. The participant is instructed to respond to target stimuli but not to respond otherwise. When a target stimulus appears, it elicits the P3b component, a positive wave within a time window of 250–500 ms over parietal brain areas (Polich, 2007) and is generally evaluated by assessing its amplitude and latency. When task-irrelevant novel or distractor stimuli are added to the oddball task, an earlier P300 component is elicited. This large positive ERP component is referred to as P3a and has a fronto-central scalp distribution.

It has been suggested that P3a and P3b reflect distinct but interrelated information processing events, with P3a reflecting initial engagement of attention, and P3b being associated with stimulus evaluation and working memory operations (Wronka, Kaiser, & Coenen, 2012). The generation of P3a is largely attributed to the prefrontal cortex, whereas P3b is thought to originate from regions including the tempo-parietal junction and the medial temporal lobes (Polich, 2007; Wronka et al., 2012).

Riby et al. (2008) investigated whether the glucose enhancement effect is restricted to neurocognitive processes related to memory. Using a crossover design, they studied the effect of a 25 mg glucose load in a group of 11 heathy young adults on different ERP components linked with attentional or memory processes using a three-stimulus visual oddball task. Specifically, they were interested in testing whether the glucose facilitation effect was related to the P3b in midline central and parietal electrode sites as an index of memory storage operations, and the P3a and P2 in frontal and central midline electrodes, to measure aspects of pre-attentive and orienting attention processes. Riby et al. (2008) observed a glucose-related decrease in P3b amplitude relative to placebo, which was associated with reduced latency and shorter duration, despite no changes in behavioural performance. The authors conclude that P3b component is sensitive to modulations in blood glucose and argue that this finding supports the sensitivity of medial temporal lobes to the glucose enhancement effect.

Knott et al. (2001) tested the effect of 50 mg glucose on ten healthy elderly adults in a repeated-measures design. They measured performance and P3b amplitude and latency during the Sternberg memory task, which is known to measure speed and efficiency of short-term storage and processing (Sternberg, 1969). No significant effect of glucose was observed on behavioural performance, nor in the amplitude or latency of the P3b response during the memory scanning task. Based on the absence of glucose related effects, the authors concluded that glucose ingestion does not alter cognitive ERPs regardless of the task involved.

The results of these two studies with respect to the P300 ERP component are not wholly consistent. One study observed neither a treatment-related effect on P3b amplitude or latency, nor on task performance (Knott et al., 2001). The other study however, observed changes in P3b amplitude, despite an absence of behavioural modulation (Riby et al., 2008). The different findings may be due to a number of factors, the most important of which is likely to be the choice of cognitive task during ERP recording. The Sternberg memory task used by Knott et al. (2001) is designed to assess storage and retrieval of random information from short-term memory, whereas the three stimulus oddball task used by Riby et al. (2008) is a selective attention task.

#### Event-Related Potentials: LP and FN400 Old-New Effects

Dual-process models of recognition propose two overlapping processes: one involving the conscious recollection of details about a previous event (‘remembering’) and the other based on familiarity with the stimulus, or ‘knowing’ (Yonelinas, 2002). These two processes have been shown to be functionally dissociable and to rely on partially separable brain regions. The hippocampus is thought to be critical for recollection, but not familiarity (Eichenbaum, Yonelinas, & Ranganath, 2007). Further, familiarity and recollection have been found to have distinct ERP correlates, namely, FN400 old-new effects and parietal late positive (LP) old-new effects, respectively (Rugg & Curran, 2007). The FN400, linked with familiarity, can be observed approximately 400–600 ms post stimulus onset as an enhanced positivity in response to old relative to new items (Rugg & Curran, 2007). Whereas the ERP linked with recollection is observed from approximately 400 ms post stimulus as a positive waveform maximal over parietal sites, known as the LP component (Curran, 2000). Three studies (Brown & Riby, 2013; Scholey et al., 2015; Smith et al., 2009) investigated the effect of glucose on ERP components thought to reflect markers of recollection (LP) and familiarity memory (FN400).

Smith et al., (2009) investigated the effect of 25 g of glucose on a recognition memory task in 17 healthy adolescent subjects using a within-subject design. The foci of this study were LP and FN400 as markers of recollection, and familiarity respectively. Participants were presented with words in an initial study phase. In the test phase participants were presented with words which had been presented in the preceding study phase (‘old’ words), words they had not studied (‘new’ words) and words that were opposite in plurality to items studied in preceding study phase (‘similar’ words). Participants were asked to press a ‘yes’ button if they remembered the presented item from the study phase and to press a ‘no’ button if not. Faster response times were reported in the glucose condition relative to placebo. Further, glucose enhanced LP effect and FN400 effect. The authors proposed that glucose administration facilitates both recollection and familiarity-based recognition memory performance, suggesting targeting of more global cortical regions.

Scholey et al. (2015) also focused on the LP recollection and the FN400 effect. The effect of 25 g glucose on neurocognitive mechanisms was tested in 12 elderly adults. ERPs were recorded during completion of a remember-know paradigm with and without an additional psychomotor tracking component. The tracking component was included to delineate the effect of task effort in the glucose facilitation. In the study phase participants were presented with words in the auditory mode. In the retrieval phase words were presented visually and participants were asked to indicate whether the word was ‘old’ (presented in the study phase) or ‘new’ (not presented in study phase). Following a ‘yes’ response, participants pressed a button indicating whether they consciously recollected the item from the study phase (‘remember’), whether the item seemed familiar but could not be recalled (‘know’) or if they were uncertain that it appeared in the study phase list (‘guess’). In the dual task version participants were asked to track a moving target during the auditory presentation in the study phase. Glucose was found to reduce overall accuracy on task performance. Further, task effort did not modulate memory enhancing effects of glucose. These findings therefore do not support task effort modulation of the glucose facilitation effect. A significantly greater amplitude of the LP effect was reported, in line with previous findings by Smith et al., (2009). In contrast to the previous study, the effect on FN400 was not significant, although it was larger in the glucose condition. This study was conducted with elderly adults and aging is associated with decline in recollection (hippocampal deficit) whereas familiarity processes stay relatively stable. The authors argue that glucose may selectively enhance processes that are known to decline in aging.

Brown and Riby (2013) investigated whether glucose modulates memory as well as other cognitive processes, particularly attention. Using a between-subjects design they compared the effect of 25 g glucose in 18 healthy participants to 17 participants who ingested a placebo. They performed an episodic memory (item recognition) task and an attention (Stroop) task while undergoing EEG recordings. The episodic memory task consisted of a study phase in which participants were presented with words and line drawings. In the test phase they were shown words and pictures they had previously studied (‘old’ items) and items they had not encountered before (‘new’ items). Glucose facilitated performance for the word condition only, which was the more difficult condition in this task. However, performance for ‘new’ items was poorer after glucose ingestion. EEG was recorded during the recognition phase and an increased LP effect was found for the more difficult (word) condition. Analysis of the ERP during episodic memory focussed on a single left parietal electrode, and thus did not report on the FN400 component. For the Stroop task, participants were presented with words of colours in different font colours, either congruent or incongruent. Participants were asked to either name the font colour or read the word. No glucose facilitation of performance was observed. For the EEG data, analysis was focused on the fronto-central negativity (350-500 ms post-stimulus, with a peak around 410-450 ms). Participants in the glucose group demonstrated a more negative-going ERP for congruent relative to incongruent stimuli – in contrast with placebo and also against the ERP pattern typically observed on the task.

Overall, studies investigating the LP and FN400 have made use of relatively similar recognition memory tasks, one of the studies added an additional tracking component during encoding to investigate the role of task effort in the glucose facilitation effect (Scholey et al., 2015), and one study also assessed ERP changes in the Stroop task (Brown & Riby, 2013). The electrophysiological studies reviewed suggest that glucose modulates recollection processes as evidenced by the modulation of LP component in the reviewed studies (Brown & Riby, 2013; Scholey et al., 2015; Smith et al., 2009). The findings relating to the FN400 familiarity component appears to be less consistent and there is limited evidence for modulation of the fronto-central activity linked with interference in the Stroop task (Brown & Riby, 2013).

Based on these ERP studies, there is some evidence that components related to working memory operations (P3b: Riby et al., 2009) and familiarity processes in recognition memory (Brown & Riby, 2013) may be modulated by glucose administration, while perhaps the most consistent evidence of neurophysiological modulation by glucose has been reported for the LP component, linked with recollection memory (Brown & Riby, 2013; Scholey et al., 2015; Smith et al., 2009).

#### Resting State EEG

Spectral analysis of resting EEG signals provides an assessment of ongoing oscillatory processes in the brain at a macroscopic level. Given the relatively strong test-retest reliability (Smit, Posthuma, Boomsma, & De Geus, 2005), spectral parameters of resting EEG have a history of use in studying pharmacological effects on the brain. The study conducted by Knott et al. (2001; described above in section 3.2.1) also acquired vigilance controlled resting EEG, which was spectral-analysed to assess tonic arousal during rest. An increase in the slow alpha frequency band (7.5–10 Hz) was observed. This increase in slow alpha frequency was interpreted as modulation of central arousal processes, however it was not reported whether these changes related to any changes in task performance.

In a resting-state EEG study including 24 healthy older adults, An et al. (2015) tested the effect of a drink containing 17 g of glucose on resting EEG spectral power, in addition to performance on two attention tests performed prior EEG recording. As a measure of verbal attention span, the digit span test (Korean adaptation of the Wechsler Adult Intelligence Scale, revised) was used, and a measure of spatial attention, spatial span (Corsi block-tapping test) was included, with forward and backward trials performed for both measures. Participants improved performance on both tests (except for the spatial span backward condition) after glucose ingestion. Further, increases in theta and low alpha bands in resting-state EEG were reported, most predominantly in frontal and posterior brain regions. These changes were not correlated with attentional performance. The testing protocol in the study conducted by An et al. (2015) showed some noteworthy differences to the other studies mentioned here, all subjects were tested after fasting first, and then 3 days later after glucose in the same order without the administration of a matched placebo drink. Without a crossover, placebo or control group, it is difficult to attribute the effect to glucose. The glucose drink was made up of orange juice containing 180 ml water mixed with 17 g of glucose. It is not clear whether the orange juice contained any additional sugars.

Results of the reviewed studies exploring glucose-related changes in resting EEG found that glucose increased spectral power in the low alpha frequency band. Further, An et al. (2015) also report an increase in theta power. These results point to changes in arousal and attentional processes, suggesting that glucose may exert its effect on more global brain processes. However, these results need replication in a more rigorously controlled study design.

### fMRI

Functional magnetic resonance imaging (fMRI) indirectly measures brain activity by detecting Blood Oxygenation Level Dependent (BOLD) signal changes associated with the haemodynamic response. Four studies used functional magnetic resonance imaging (fMRI) to assess the glucose facilitation effect. Two studies examined the effect of glucose on episodic memory performance with a special focus on task-related activity during the episodic memory encoding (Parent et al., 2011; Stone et al., 2005).

Stone et al. (2005) tested seven medicated adults diagnosed with schizophrenia in a repeated-measures design in which patients consumed 50 g glucose during a verbal encoding and recognition memory task known to elicit increased temporal lobe activity. Participants performed the verbal encoding task while undergoing fMRI scanning. Compared to the placebo condition, greater activation of left parahippocampus was observed during novel sentence encoding, despite no change in memory performance. Stone and colleagues’ study (2005) was a pilot study and has several limitations that should be noted. The study had a very limited sample size (*n* = 7). Furthermore, the subjects in this study were diagnosed with chronic schizophrenia who were all medicated, although standardized for medications, which could lead to unintended drug interactions with the experimental outcome measures.

Parent et al. (2011) analysed fMRI data of twelve healthy male participants, who were given 50 g glucose or placebo in randomized order. Participants completed a picture encoding task in the scanner. Upon completion of the MRI scan, participants’ memory for the picture stimuli was assessed with a five-minute delay, free-recall task, and again 1 day later. A series of analyses to examine the effects of glucose on brain activity were conducted. The effect of glucose on activation changes during encoding were examined and an increase in the superior parietal gyrus was reported. Further, the effects of glucose on encoding related to successful subsequent episodic memory (as assessed at both five-minute and 1 day delays) were examined employing the difference in subsequent memory (DM) procedure. For the five-minute delay recall test glucose was associated with increased subsequent memory-related encoding activation in the precuneus, supramarginal gyrus and paracentral lobule. A decrease was reported in the right rolandic operculum. For the one-day delay, increased activation was reported in the right hippocampus, middle frontal gyrus and inferior parietal cortex. Decreases were reported in precentral and post-central gyri as well as left inferior temporal gyrus.

Parent et al. (2011) also explored task-related functional connectivity analyses using left and right hippocampus and left and right amygdala as seed regions of interests (ROI). Glucose relative to placebo increased functional connectivity of left and right hippocampus with numerous brain regions in bilateral temporal and prefrontal cortices, as well as bilateral insula and fusiform gyri. Further, the right hippocampus showed increased connectivity with left hippocampus, right parahippocampal gyrus and bilateral amygdala. Left and right amygdala seed regions showed increased functional connectivity with several prefrontal and temporal regions, including the contralateral hippocampus after glucose administration. These effects differed for negative versus neutral stimuli, negative pictures elicited greater activation in several limbic, prefrontal and parietal regions, whereas neutral pictures evinced activation in fewer regions.

Both Stone et al. (2005) and Parent et al. (2011) focused on the domain of episodic memory. Whereas Stone et al. tested a small clinical sample, Parent et al. (2011) studied a group of healthy male adults. Stone et al. (2005) made use of a verbal encoding and recognition task, whereas Parent et al. (2011) used a picture encoding and recall task with 5 min and one-day delay and further investigated the influence of emotional valence of the pictures. Both studies reported increases in activation during the encoding phase albeit in different regions. Whereas Stone et al. (2005) reported increase in activation of left posterior parahippocampus during encoding, Parent et al. (2011) reported an increase in the superior parietal gyrus in the encoding phase. Parent et al. (2011) also examined changes in functional connectivity during encoding. When using the difference in subsequent memory (DM) Parent et al. (2011) found changes in brain activity related to successful subsequent episodic memory (with a 5 min and a 1 day delay), which included the right hippocampus and associated frontal and parietal regions.

Serra-Grabulosa et al. (2010) investigated the effect of glucose and caffeine, both combined and in isolation, on sustained attention using a continuous performance task. In a parallel groups, double-blind, randomized design, participants were allocated to one of four treatment drink conditions comprising placebo (150 ml water), glucose (75 g of glucose), caffeine (75 mg of caffeine) and glucose and caffeine combined (75 g glucose +75 mg of caffeine). There were ten participants per arm and groups were gender balanced. They underwent two MRI scanning sessions in 1 day, one following an overnight fast (at least 8 h) and one after the ingestion of the beverage. During the scan, participants performed a sustained attention task, the continuous performance test (CPT-IP). Here we only focus on the glucose arm of the study. Task performance was the same before and after ingestion of the beverage. No differences in functional brain activity, as measured in BOLD signal change, were observed. This study had a few limitations that should be noted. The use of parallel groups with scanning sessions pre-and post-ingestion can introduce uncontrolled effects of repeated exposure to task. Furthermore, sample size was limited with ten participants per group and participants did not receive a matched placebo drink.

Zanchi et al. (2018) conducted a pilot study with 12 participants in which glucose, fructose and placebo were administered through a nasogastric tube in randomized order on three study visits. At the glucose visit, the drink consisted of 75 g glucose dissolved in 300 ml water and on the placebo visit the treatment was plain water. There was also a fructose arm to the study that will not be discussed for this review. Participants underwent MRI scanning and completed a working memory task (n-back) and an attentional control task (Go/No-Go) in the scanner as well as a resting state scan. No behavioural change in performance was observed. However, compared to placebo, glucose decreased task-related activity for both n-back and Go/No-Go task in frontal regions, specifically in the anterior cingulate cortex and dorsolateral prefrontal cortex. Analysis of the resting state data revealed that resting state connectivity within the frontoparietal and salience networks increased. The decrease in task-related activity has been interpreted as showing increased neural efficiency in response to cognitive task after glucose compared to placebo (Erickson et al., 2005). Increases in functional connectivity after glucose ingestion have been reported before (Page et al., 2013; Wölnerhanssen et al., 2015). These studies however, did not report cognitive outcome measures and were excluded from the current review.

#### Discussion of fMRI Findings

Overall the studies using fMRI to study the glucose facilitation effect suggest that glucose changes regional brain activity (Parent et al., 2011; Stone et al., 2005) and functional connectivity (Parent et al., 2011) during encoding. Further, glucose influenced brain regions in terms of regional activation related to encoding success, as measured at short and longer delays (Parent et al., 2011). It is noteworthy that these studies described the effect of glucose on encoding processes, however they did not report significant effects of glucose on memory performance. Whether glucose also effects episodic retrieval processes remains unclear. Further replication of studies with more challenging memory tasks and tasks involving divided attention at encoding are suggested.

Whereas the studies focusing on episodic memory encoding reported increases in task related activity following glucose ingestion, the study that focused on tasks related to working memory and attentional control reported decreases in task related activity in frontal regions for both tasks (Zanchi et al., 2018). Further, the available fMRI evidence does not support an effect of glucose on brain activity assessed during sustained attention (Serra-Grabulosa et al., 2010). The increase of brain activity engaged during episodic paradigms has been interpreted as beneficial increases in encoding processes (Parent et al., 2011), whereas the decreases in working memory and attentional control task related activity reported by Zanchi et al. have been interpreted as evidence of neural efficiency during the task.

The two studies that examined functional connectivity after glucose ingestion compared to placebo, both reported increases in connectivity in the glucose elevated state (Parent et al., 2011; Zanchi et al., 2018). The study that investigated encoding-related connectivity with a hypothesis-driven, seed-based analysis observed increases in functional connectivity to regions related to episodic memory (Parent et al., 2011). The other study investigated functional connectivity in the resting state using a data-driven approach (ICA: Zanchi et al., 2018). Increases in functional connectivity in the frontoparietal and salience networks were reported after glucose ingestion compared to placebo.

### fNIRS

The use of functional Near-Infrared Spectroscopy (fNIRS) relies (as fMRI) on the BOLD response, albeit typically at a lower resolution. This non-invasive imaging technique uses near-infrared light to examine the function of the brain. A single study has utilised fNIRS to explore the glucose facilitation effect (Gagnon et al., 2012). They examined 15 elderly, non-diabetic participants in a double-blind, placebo-controlled, crossover trial. The participants were given 50 g of glucose in the active condition and performed an attentional dual-task paradigm while event-related fNIRS was recorded. In the glucose condition, participants showed similar dual-task costs for both tasks, whereas in the placebo condition participants prioritized one task over the other, with a significantly larger dual-task cost for the non-prioritized task. Differential brain activation was also observed in right ventral–lateral prefrontal regions for oxygenated hemoglobin and deoxygenated hemoglobin, with more activation apparent in the glucose condition. The authors concluded that glucose ingestion may temporarily enhance the capacity to coordinate two concurrent tasks equally in healthy elderly adults as reflected in brain activation patterns. Replication of results of this study are needed as the analyses were run for each sensor separately and were not controlled for multiple comparisons.

## Discussion

To our knowledge, this is the first systematic review of studies exploring the effects of exogenous glucose on cognition and brain function using neuroimaging methods. Eleven studies satisfied the inclusion criteria of including cognitive as well as neuroimaging tests. The reviewed studies differed widely with respect to cognitive task and experimental design, imaging modalities used, participant groups and sample sizes.

### Reported Effects on Cognitive Performance

Of the eleven studies reviewed here, only five studies reported a significant modulation of cognitive outcome measures. One study reported faster response times after the ingestion of glucose in a recognition memory task (Smith et al., 2009), another reported greater accuracy during an episodic memory task and a trend towards improved performance on an attentional test (Brown & Riby, 2013). One study reported improvements in performance on an attention task (An et al., 2015) and another reported improvements in the ability to efficiently coordinate concurrent tasks in an attentional control task (Gagnon et al., 2012). Interestingly, one study reported reduced overall accuracy in a remember-know paradigm after glucose (Scholey et al., 2015).

The other six studies included in this review did not report any significant modulation on task performance. Based on the vast amount of behavioural research investigating the glucose facilitation effect (see e.g. Smith et al., 2011 for review), the lack of behavioural modulation may appear surprising. However, this is possibly due to the relatively small sample sizes of the included neuroimaging studies, resulting in sub-optimal statistical power to detect the cognitive performance changes.

### Reported Effects on Neurophysiological and Neuroimaging Markers

With the exception of one study (Serra-Grabulosa et al., 2010), all studies included in this review, reported modulation of some neurophysiological or neuroimaging markers with glucose administration. The evidence from studies investigating ERP effects are mixed. One study reported a reduction of P3b amplitude after glucose (Riby et al., 2008) whereas the other did not find changes in the same measure (Knott et al., 2001). This literature provides limited evidence for glucose effects on stimulus evaluation and working memory operations (as indexed by P3b amplitude). It is worth noting that the two studies used different task paradigms to elicit the P3b (an oddball paradigm and the Sternberg memory task), raising the possibility that methodological differences may contribute to these differences in glucose-related effects.

Of the two studies to report on glucose modulation of the familiarity-related FN400 in recognition tasks, one in adolescents observed an increase (Smith et al., 2009), while the study of Scholey et al. (2015) observed a marginal trend in the same direction in older adults. There is some evidence to suggest frontal familiarity-related ERPs may not follow adult patterns in children (Czernochowski, Mecklinger, & Johansson, 2009). The somewhat inconsistent findings of FN400 modulation by glucose across the two studies in a younger and older sample, when combined with the fact that the FN400 component was not prominent in the placebo condition of Smith et al., suggest a need for further work to fully characterise the potential effects of glucose administration familiarity-related FN400 electrophysiological signal.

In contrast, the three studies investigating the LP component through episodic memory paradigms reported a largely consistent increase in the LP component with glucose administration (Brown & Riby, 2013; Scholey et al., 2015; Smith et al., 2009). These results support the findings of a range of behavioural studies (see Smith et al., 2011 for review) reporting improvements especially in the domain of episodic memory. Glucose increased the LP component, which is a component linked with recollection, which could be interpreted as supporting the notion that glucose enhances neuro-cognitive processes related to episodic memory. It is also noteworthy that two of these studies further reported the modulation of attentional ERPs outside the episodic memory paradigms (Brown & Riby, 2013; Riby et al., 2008), partially arguing against the specificity of the effect. Further evidence for modulation of attention and arousal processes stems from the spectral analysis of resting EEG in two studies which revealed increases of alpha power (An et al., 2015; Knott et al., 2001), and an increase in theta band power (An et al., 2015).

The available evidence from fMRI studies suggests that increasing glucose levels can modulate hemodynamic response during completion of cognitive tasks. Two fMRI studies (Parent et al., 2011; Stone et al., 2005) reported increased activity in the medial temporal lobes after glucose administration during learning and memory tasks. Specifically, enhanced activation of the left parahippocampus was observed during encoding under glucose in one study (Stone et al., 2005). In another study, glucose administration increased activity in brain regions associated with episodic memory encoding and regions associated with subsequent successful recall (including the hippocampus and prefrontal cortex), and further modulated functional connectivity between hippocampus, amygdala and a series of other brain regions related to successful encoding (Parent et al., 2011). Whilst these two episodic encoding fMRI studies observe somewhat overlapping results, it is also important to note the different samples (patients with schizophrenia and non-clinical adult males) and different task designs (verbal encoding and emotional picture recognition). Another interesting divergence in research paradigms was observed between fMRI and EEG studies, whereby the fMRI studies investigating declarative memory processes focused on activation changes during encoding (Parent et al., 2011; Stone et al., 2005), ERP research has focussed on retrieval (Brown & Riby, 2013; Scholey et al., 2015; Smith et al., 2009). A more recently published study has reported differential, age-related changes in resting state hippocampal connectivity in response to glucose. Functional connectivity changes were correlated with both glycemic response and (out of scanner) spatial learning performance (Peters et al., 2020). 

Beyond episodic memory, one fMRI study (Zanchi et al., 2018) demonstrated decreases in activation during working memory and response inhibition tasks in frontal regions (anterior cingulate cortex and dorsolateral prefrontal cortex) and increases in connectivity within the frontoparietal and salience network. Additional support for the modulation of frontal regions after glucose ingestion stems from the fNIRS study, albeit with evidence of increased activity during a dual-task paradigm (Gagnon et al., 2012). The fMRI study focusing on the domain of sustained attention, did not report any modulation of brain activity in response to glucose (Serra-Grabulosa et al., 2010). Taken together, the present review provides some support consistent with evidence from previous behavioural investigations, that there are certain brain regions more sensitive to increased peripheral glucose, specifically in the medial temporal lobe and frontal areas, and networks. Furthermore, the reviewed studies have reported these modulations in neurophysiology across cases where behavioural performance has been improved and others where this has not been observed.

### Methodological Considerations

The variability in experimental methodologies and reporting methods complicates the interpretation of the studies reviewed. Reporting standards of the included studies ranged from four to eight (out of ten) on the modified Jadad scale. Comprehensive reporting of methodologies is essential for replication and interpretation. The research field investigating the glucose facilitation effect can be regarded as bridging a gap between cognitive neuroscience and clinical trials research, with the issues in reporting standards perhaps reflective of these issues in cognitive neuroscience and clinical trial reporting. The field is undergoing a renewed focus on replication of experimental results and increasing the quality of experiments (e.g. increasing sample sizes), improving reporting practices and transparency of data analysis and data availability (Carp, 2012; Yarkoni, Poldrack, Van Essen, & Wager, 2010).

Besides improving reporting standards, there are other sources of variability in methodological choices that render the interpretation of results of the reviewed studies complicated. One important consideration is the general experimental design. Within-subject designs for these kinds of studies are more powerful than between-subject designs. However, it can be argued that within-subject designs carry the potential for carry-over effects, that is, practice effects. To counteract the risk of learning effects the order of drinks should be counterbalanced, and parallel versions of cognitive tests should be used. It is thus important to consider randomized, double-blind, counterbalanced, within-subject designs in future investigations of the glucose facilitation effect.

A potential source of variability in the study of glucose on cognition is the amount administered. Of the studies included in this review, four used 25 g, four administered 50 g, two used 75 g and one study reported 17 g of glucose. There is evidence that the glucose facilitation effect follows an inverted U-shape dose-response curve in humans (Owen, Scholey, Finnegan, Hu, & Sünram-Lea, 2012; Parsons & Gold, 1992), with 25 g being reported as the optimal dose to be administered for memory enhancement effect (Riby, 2004), while decreases in performance have been observed after administration of 75 g (Parsons & Gold, 1992). Furthermore, the influence of acute glucose load is time-dependent (Gold, 1986). Despite the importance of timing in studying the effect of fluctuating glucose levels, few of the studies reviewed specified the time frame, relative to glucose loading, in which testing took place.

Another factor to consider is pre-trial fast duration. Most studies included in this review were conducted in the morning after an overnight fast. The glucose facilitation effect has also been demonstrated after a 2 h fasting period in young adults (Sünram-Lea, Owen, Finnegan, & Hu, 2011). Three studies included in this review were conducted after a 2-h fasting period (Brown & Riby, 2013; Riby et al., 2008; Smith et al., 2009). All of these studies reported modulations of neurophysiological markers, and all but one of these (Riby et al., 2008) also reported improvements in task performance. This has important implications for examining the effect in more naturalistic settings (Riby et al., 2017).

A number of factors have been proposed that may confound the study outcomes. These factors should be considered in the study inclusion and exclusion criteria or should be statistically adjusted for, and include age, gender, body weight, and glucoregulatory efficiency (Kaplan, Greenwood, Winocur, & Wolever, 2000). Sex differences have been reported in glucoregulatory response (Craft, Murphy, & Wemstrom, 1994; Paula et al., 1990). To mitigate these effects, Parent et al. (2011) and Zanchi et al. (2018) recruited only male participants for their studies, none of the other studies accounted for sex differences.

Individual differences in glucose regulation may interact with the cognitive improvements after glucose ingestion (Riby, 2004). Research has shown that glucose regulatory efficiency, that is, the efficiency with which supplemental glucose is cleared from the blood, becomes less efficient as people get older, and has been correlated with performance on tasks probing attentional control in elderly adults (Gagnon, Greenwood, & Bherer, 2011). Therefore, glucose regulation is a factor that should be included in the analysis especially in the study of aging. Only three studies considered individual glucoregulatory efficiency in their analyses (Brown & Riby, 2013; Gagnon et al., 2012; Knott et al., 2001).

Interestingly, only six of the reported studies investigated the effect in healthy young adults (An et al., 2015; Brown & Riby, 2013; Parent et al., 2011; Riby et al., 2008; Serra-Grabulosa et al., 2010). None of these were tested in the same modality or with the same tasks. In order to characterise the glucose facilitation effect in healthy adults, more studies in this group are needed, with a strong need for replication of previous findings. The other age groups that were investigated (adolescents and elderly adults) are difficult to interpret without a comparison to a control group. In order to interpret findings of adolescents, elderly or patient populations (e.g. patients with schizophrenia), the effect should be characterised in healthy control groups. This way, findings in clinical or at-risk populations can be benchmarked against the effects observed in healthy adults.

Taken together, the glucose facilitation effect has been studied using a range of neuroimaging methods with highly variable designs. The understanding of these effects will be enhanced by the application of consistent paradigms tested in various imaging modalities in order to determine the neural underpinnings of the effect. A key challenge for integrating this literature is the heterogeneity of the cognitive paradigms adopted. Even where episodic memory paradigms are considered in fMRI and EEG studies, the fMRI studies focused on activation changes during encoding and ERP research focussed on retrieval or recognition.

### Summary of Neuroimaging Methods

The systematic literature search identified studies utilising primarily fMRI and EEG as the neuroimaging modalities, with one study using fNIRS. The temporal resolution of EEG methods allows exploring stages of processing which may be preferentially affected after glucose manipulation. As a direct measure of brain electrical activity, these methods have the advantage of not having potential confounding by non-neural factors that influence haemodynamic measures (e.g. blood flow). However, these EEG methods are less well suited to localizing changes in activity and are particularly challenging for deeper structures such as those of interest to this research field. This limitation is somewhat minimized in magnetoencephalography (MEG), which may be a method for future work to pursue regional hypotheses without potential haemodynamic confounds and maintaining the capacity to isolate processing stages.

fNIRS is another cost-effective measure with a good temporal resolution. It has a few limitations that need to be accounted for: it has only a low cerebral penetration depth, and enables researchers only to study cortical areas (Mehagnoul-Schipper et al., 2002) and localisation of activated regions is difficult due to low spatial resolution. In the study of the glucose facilitation effect, fMRI has helped further implicate the medial temporal lobes in this effect. Since temporal resolution of fMRI does not allow study of the rich temporal dynamics of the underlying electrophysiological activity (Logothetis, 2008) using multi-modal methodologies may also offer an important opportunities to reveal the mechanisms supporting these effects.

An interesting modality to consider for future studies is proton magnetic resonance spectroscopy (^1^H MRS), a non-invasive technique that measures neurometabolites in vivo (Cleeland, Pipingas, Scholey, & White, 2019). Haley et al. (2006) reported increased glucose concentration in the hippocampus following oral glucose administration. This study used a pre-post paradigm with each participant serving as their own control. Measurements were taken from the right hippocampal region and results showed that individuals with Alzheimer’s disease (AD) had a significant increase in cerebral glucose concentration in the hippocampus following oral glucose administration, while healthy older adults did not. These findings were interpreted in line with the notion that individuals with AD have impaired glucoregulation, consistent with prior PET studies showing this hypometabolism in AD individuals (Herholz, 2003). There remain complexities in quantifying glucose via ^1^H MRS, as the multiple resonances each have spectral overlap with other metabolites or water. Future research should aim to investigate individual differences in MRS signal and correlate these with cognitive outcome measures.

Another recently developed non-invasive MR technique that has demonstrated to be sensitive to glucose administration is measuring dynamic changes in cerebral metabolic rate of oxygen consumption (CMRO_2_). Xu et al. (2015) used a method known as T_2_-relaxation-under-spin-tagging (TRUST) to measure CMRO_2_ after the ingestion of 50 g of glucose, compared with a control group that was scanned without ingestion of glucose (*n* = 10 in both groups). In the 40 min following glucose ingestion, CMRO_2_ decreased and oxygen extraction fraction was reduced, while cerebral blood flow was unchanged. In the control group no changes for CMRO_2_, cerebral blood flow and oxygen extraction fraction were observed. These findings provide the interesting suggestions that acutely administered glucose may decrease the global rate of cerebral metabolism of oxygen at rest. As with MRS, the relevance of this measure to cognition is yet to be explored, and these two methods offer interesting avenues for future research.

## Conclusion

A systematic review was conducted to explore the neural correlates of the glucose facilitation effect. Eleven studies were identified that investigated the effect of increased peripheral blood glucose on cognition and neurocognitive markers using in a range of modalities. Only five studies reported modulation of behavioural performance on cognitive tasks, however ten out of eleven studies reported modulation of neurocognitive markers. The nature of the studies included in this review is very diverse, with respect to methodologies used, participant samples (age groups, etc.) as well as tasks used to elicit neuroimaging markers. Medial temporal and frontal brain areas were reported to be affected depending on the task paradigms adopted.

The area would certainly benefit from more studies incorporating neuroimaging measures at different phases of task performance, such as during encoding and retrieval in episodic memory tasks, to understand whether distinct components of a task are specifically influenced by glucose. Further, careful design of functional imaging task paradigms to explore the specificity of the glucose facilitation effect, for example manipulating task load and task strategies in an effort to isolate the proposed mechanisms may offer further insights.

Taken together, the evidence from neuroimaging studies exploring the glucose facilitation of cognitive performance is limited not only by the relatively small number of studies but also by identified methodological issues which need to be addressed in future studies.

## References

[CR1] An YJ, Jung K-Y, Kim SM, Lee C, Kim DW (2015). Effects of blood glucose levels on resting-state EEG and attention in healthy volunteers. Journal of Clinical Neurophysiology.

[CR2] Benton D, Owens DS, Parker PY (1994). Blood glucose influences memory and attention in young adults. Neuropsychologia.

[CR3] Benton D, Stevens MK (2008). The influence of a glucose containing drink on the behavior of children in school. Biological Psychology.

[CR4] Brandt KR, Gibson EL, Rackie JM (2013). Differential facilitative effects of glucose administration on Stroop task conditions. Behavioral Neuroscience.

[CR5] Brown LA, Riby LM (2013). Glucose enhancement of event-related potentials associated with episodic memory and attention. Food & Function.

[CR6] Carp J (2012). The secret lives of experiments: Methods reporting in the fMRI literature. Neuroimage.

[CR7] Cleeland, C., Pipingas, A., Scholey, A., & White, D. (2019). Neurochemical changes in the aging brain: A systematic review. *Neuroscience & Biobehavioral Reviews*. 10.1016/j.neubiorev.2019.01.003 30625337

[CR8] Craft S, Murphy C, Wemstrom J (1994). Glucose effects on complex memory and nonmemory tasks: The influence of age, sex, and glucoregulatory response. Psychobiology.

[CR9] Craft S, Watson GS (2004). Insulin and neurodegenerative disease: Shared and specific mechanisms. The Lancet. Neurology.

[CR10] Curran T (2000). Brain potentials of recollection and familiarity. Memory & Cognition.

[CR11] Czernochowski D, Mecklinger A, Johansson M (2009). Age-related changes in the control of episodic retrieval: An ERP study of recognition memory in children and adults. Developmental Science.

[CR12] Dore S, Kar S, Rowe W, Quirion R (1997). Distribution and levels of [125I] IGF-I,[125I] IGF-II and [125I] insulin receptor binding sites in the hippocampus of aged memory-unimpaired and-impaired rats. Neuroscience.

[CR13] Eichenbaum H, Yonelinas AP, Ranganath C (2007). The medial temporal lobe and recognition memory. Annual Review of Neuroscience.

[CR14] Erickson KI, Colcombe SJ, Wadhwa R, Bherer L, Peterson MS, Scalf PE, Kramer AF (2005). Neural correlates of dual-task performance after minimizing task-preparation. Neuroimage.

[CR15] Gagnon C, Desjardins-Crepeau L, Tournier I, Desjardins M, Lesage F, Greenwood CE (2012). Near-infrared imaging of the effects of glucose ingestion and regulation on prefrontal activation during dual-task execution in healthy fasting older adults. Behavioural Brain Research.

[CR16] Gagnon C, Greenwood CE, Bherer L (2011). Glucose regulation is associated with attentional control performances in nondiabetic older adults. Journal of Clinical and Experimental Neuropsychology.

[CR17] Gailliot MT, Baumeister RF (2007). The physiology of willpower: Linking blood glucose to self-control. Personality and Social Psychology Review.

[CR18] Gold PE (1986). Glucose modulation of memory storage processing. Behavioral and Neural Biology.

[CR19] Haley, A. P., Knight-Scott, J., Simnad, V. I., & Manning, C. A. (2006). Increased glucose concentration in the hippocampus in early Alzheimer’s disease following oral glucose ingestion. *Magnetic Resonance Imaging, 24*(6), 715–720. 10.1016/j.mri.2005.12.020 16824966

[CR20] Herholz K (2003). PET studies in dementia. Annals of Nuclear Medicine.

[CR21] Horne P, Barr RG, Valiante G, Zelazo PR, Young SN (2006). Glucose enhances newborn memory for spoken words. Developmental Psychobiology: The Journal of the International Society for Developmental Psychobiology.

[CR22] Hoyland A, Lawton CL, Dye L (2008). Acute effects of macronutrient manipulations on cognitive test performance in healthy young adults: A systematic research review. Neuroscience & Biobehavioral Reviews.

[CR23] Kaplan RJ, Greenwood CE, Winocur G, Wolever TM (2000). Cognitive performance is associated with glucose regulation in healthy elderly persons and can be enhanced with glucose and dietary carbohydrates. The American Journal of Clinical Nutrition.

[CR24] Kennedy DO, Scholey AB (2000). Glucose administration, heart rate and cognitive performance: Effects of increasing mental effort. Psychopharmacology.

[CR25] Knott V, Messier C, Mahoney C, Gagnon M (2001). Glucose and glucoregulatory modulation of memory scanning, event-related potentials and EEG in elderly subjects. Neuropsychobiology.

[CR26] Logothetis NK (2008). What we can do and what we cannot do with fMRI. Nature.

[CR27] Macpherson H, Roberstson B, Sünram-Lea S, Stough C, Kennedy D, Scholey A (2015). Glucose administration and cognitive function: Differential effects of age and effort during a dual task paradigm in younger and older adults. Psychopharmacology.

[CR28] Magistretti, P. J. (1999). Brain energy metabolism. Academic Press.

[CR29] Manning CA, Ragozzino ME, Gold PE (1993). Glucose enhancement of memory in patients with probable senile dementia of the Alzheimer's type. Neurobiology of Aging.

[CR30] McEwen BS, Reagan LP (2004). Glucose transporter expression in the central nervous system: Relationship to synaptic function. European Journal of Pharmacology.

[CR31] Mehagnoul-Schipper DJ, van der Kallen BF, Colier WN, van der Sluijs MC, van Erning LJTO, Thijssen HO (2002). Simultaneous measurements of cerebral oxygenation changes during brain activation by near-infrared spectroscopy and functional magnetic resonance imaging in healthy young and elderly subjects. Human Brain Mapping.

[CR32] Messier C (2004). Glucose improvement of memory: A review. European Journal of Pharmacology.

[CR33] Messier C, Gagnon M, Knott V (1997). Effect of glucose and peripheral glucose regulation on memory in the elderly. Neurobiology of Aging.

[CR34] Miller HC, Bourrasseau C, Blampain J (2013). Can you enhance executive control without glucose? The effects of fructose on problem solving. Journal of Psychopharmacology.

[CR35] Moher D, Liberati A, Tetzlaff J, Altman DG, Group, P (2009). Preferred reporting items for systematic reviews and meta-analyses: The PRISMA statement. PLoS Medicine.

[CR36] Owen L, Scholey A, Finnegan Y, Sünram-Lea SI (2013). Response variability to glucose facilitation of cognitive enhancement. British Journal of Nutrition.

[CR37] Owen L, Scholey AB, Finnegan Y, Hu H, Sünram-Lea SI (2012). The effect of glucose dose and fasting interval on cognitive function: A double-blind, placebo-controlled, six-way crossover study. Psychopharmacology.

[CR38] Page KA, Chan O, Arora J, Belfort-DeAguiar R, Dzuira J, Roehmholdt B (2013). Effects of fructose vs glucose on regional cerebral blood flow in brain regions involved with appetite and reward pathways. Jama.

[CR39] Parent MB, Krebs-Kraft DL, Ryan JP, Wilson JS, Harenski C, Hamann S (2011). Glucose administration enhances fMRI brain activation and connectivity related to episodic memory encoding for neutral and emotional stimuli. Neuropsychologia.

[CR40] Parsons MW, Gold PE (1992). Glucose enhancement of memory in elderly humans: An inverted-U dose-response curve. Neurobiology of Aging.

[CR41] Paula F, Pimenta W, Saad M, Paccola G, Piccinato C, Foss M (1990). Sex-related differences in peripheral glucose metabolism in normal subjects. Diabete & metabolisme.

[CR42] Peters, R., White, D. J., Cornwell, B. R., & Scholey, A. (2020). Functional connectivity of the anterior and posterior hippocampus: differential effects of glucose in younger and older adults. *Frontiers in Aging Neuroscience, 12*, 8. 10.3389/fnagi.2020.00008 10.3389/fnagi.2020.00008 PMC7004964 32082138

[CR43] Polich J (2007). Updating P300: An integrative theory of P3a and P3b. Clinical Neurophysiology.

[CR44] Riby L, Marriott A, Bullock R, Hancock J, Smallwood J, McLaughlin J (2009). The effects of glucose ingestion and glucose regulation on memory performance in older adults with mild cognitive impairment. European Journal of Clinical Nutrition.

[CR45] Riby, L., & Riby, D. (2006). Glucose, ageing and cognition: The hippocampus hypothesis.

[CR46] Riby LM (2004). The impact of age and task domain on cognitive performance: A meta-analytic review of the glucose facilitation effect. Brain Impairment.

[CR47] Riby LM, Ong DLT, Azmie NBM, Ooi EL, Regina C, Yeo EKW (2017). Impulsiveness, postprandial blood glucose, and glucoregulation affect measures of behavioral flexibility. Nutrition Research.

[CR48] Riby, L. M., Sünram-Lea, S. I., Graham, C., Foster, J. K., Cooper, T., Moodie, C., et al. (2008). P3b versus P3a: An event-related potential investigation of the glucose facilitation effect. *Journal of Psychopharmacology*, *22*(5), 486–492. 10.1177/0269881107081561 10.1177/0269881107081561 18208932

[CR49] Rugg MD, Curran T (2007). Event-related potentials and recognition memory. Trends in Cognitive Sciences.

[CR50] Sarris J, Byrne GJ (2011). A systematic review of insomnia and complementary medicine. Sleep Medicine Reviews.

[CR51] Scholey, A. (2001). Fuel for thought. *The Psychologist*, 196–201.

[CR52] Scholey A, Camfield D, Macpherson H, Owen L, Nguyen P, Stough C (2015). Hippocampal involvement in glucose facilitation of recognition memory: Event-related potential components in a dual-task paradigm. Nutrition and Aging.

[CR53] Scholey A, Macpherson H, Sünram-Lea S, Elliott J, Stough C, Kennedy D (2013). Glucose enhancement of recognition memory: Differential effects on effortful processing but not aspects of ‘remember-know’responses. Neuropharmacology.

[CR54] Scholey A, Owen L (2013). Effects of chocolate on cognitive function and mood: A systematic review. Nutrition Reviews.

[CR55] Scholey, A. B., Benson, S., Sela-Venter, S., Mackus, M., & Moss, M. C. (2019). Oxygen administration and acute human cognitive enhancement: Higher cognitive demand leads to a more rapid decay of transient hyperoxia. *Journal of Cognitive Enhancement*, 1–6.

[CR56] Scholey AB, Harper S, Kennedy DO (2001). Cognitive demand and blood glucose. Physiology & Behavior.

[CR57] Scholey AB, Laing S, Kennedy DO (2006). Blood glucose changes and memory: Effects of manipulating emotionality and mental effort. Biological Psychology.

[CR58] Scholey AB, Sünram-Lea SI, Greer J, Elliott J, Kennedy DO (2009). Glucose administration prior to a divided attention task improves tracking performance but not word recognition: Evidence against differential memory enhancement?. Psychopharmacology.

[CR59] Serra-Grabulosa JM, Adan A, Falcon C, Bargallo N (2010). Glucose and caffeine effects on sustained attention: An exploratory fMRI study. Human Psychopharmacology.

[CR60] Smit D, Posthuma D, Boomsma D, De Geus E (2005). Heritability of background EEG across the power spectrum. Psychophysiology.

[CR61] Smith MA, Foster JK (2008). Glucoregulatory and order effects on verbal episodic memory in healthy adolescents after oral glucose administration. Biological Psychology.

[CR62] Smith, M. A., Riby, L. M., Eekelen, J. A., & Foster, J. K. (2011). Glucose enhancement of human memory: A comprehensive research review of the glucose memory facilitation effect. *Neuroscience and Biobehavioral Reviews*, *35*(3), 770–783. 10.1016/j.neubiorev.2010.09.008 10.1016/j.neubiorev.2010.09.008 20883717

[CR63] Smith, M. A., Riby, L. M., Sünram-Lea, S. I., Van Eekelen, J., & Foster, J. K. (2009). Glucose modulates event-related potential components of recollection and familiarity in healthy adolescents. *Psychopharmacology*, *205*(1), 11–20. 10.1007/s00213-009-1509-4 19274454

[CR64] Sternberg S (1969). Memory-scanning: Mental processes revealed by reaction-time experiments. American Scientist.

[CR65] Stone WS, Seidman LJ, Wojcik JD, Green AI (2003). Glucose effects on cognition in schizophrenia. Schizophrenia Research.

[CR66] Stone WS, Thermenos HW, Tarbox SI, Poldrack RA, Seidman LJ (2005). Medial temporal and prefrontal lobe activation during verbal encoding following glucose ingestion in schizophrenia: A pilot fMRI study. Neurobiology of Learning and Memory.

[CR67] Sünram-Lea, S. I., Dewhurst, S. A., & Foster, J. K. (2008). The effect of glucose administration on the recollection and familiarity components of recognition memory. *Biological Psychology*, *77*(1), 69–75. 10.1016/j.biopsycho.2007.09.006 10.1016/j.biopsycho.2007.09.006 17950982

[CR68] Sünram-Lea SI, Foster JK, Durlach P, Perez C (2002). Investigation into the significance of task difficulty and divided allocation of resources on the glucose memory facilitation effect. Psychopharmacology.

[CR69] Sünram-Lea SI, Owen L (2017). The impact of diet-based glycaemic response and glucose regulation on cognition: Evidence across the lifespan. Proceedings of the Nutrition Society.

[CR70] Sünram-Lea SI, Owen L, Finnegan Y, Hu H (2011). Dose–response investigation into glucose facilitation of memory performance and mood in healthy young adults. Journal of Psychopharmacology.

[CR71] van der Zwaluw NL, van de Rest O, Kessels RP, de Groot LC (2015). Effects of glucose load on cognitive functions in elderly people. Nutrition Reviews.

[CR72] Wölnerhanssen, B. K., Meyer-Gerspach, A. C., Schmidt, A., Zimak, N., Peterli, R., Beglinger, C., et al. (2015). Dissociable behavioral, physiological and neural effects of acute glucose and fructose ingestion: A pilot study. *PLoS One*, *10*(6), e0130280. 10.1371/journal.pone.0130280 10.1371/journal.pone.0130280 PMC4481317 26107810

[CR73] Wronka, E., Kaiser, J., & Coenen, A. M. (2012). Neural generators of the auditory evoked potential components P3a and P3b. 10.55782/ane-2012-1880 22508084

[CR74] Xu F, Liu P, Pascual JM, Xiao G, Huang H, Lu H (2015). Acute effect of glucose on cerebral blood flow, blood oxygenation, and oxidative metabolism. Human Brain Mapping.

[CR75] Yarkoni T, Poldrack RA, Van Essen DC, Wager TD (2010). Cognitive neuroscience 2.0: Building a cumulative science of human brain function. Trends in Cognitive Sciences.

[CR76] Yonelinas AP (2002). The nature of recollection and familiarity: A review of 30 years of research. Journal of Memory and Language.

[CR77] Zanchi D, Meyer-Gerspach AC, Schmidt A, Suenderhauf C, Depoorter A, Drewe J (2018). Acute effects of glucose and fructose administration on the neural correlates of cognitive functioning in healthy subjects: A pilot study. Frontiers in Psychiatry.

